# Multimodal MEMPRAGE, FLAIR, and ${\text{R}}_{2}^{*}$ Segmentation to Resolve Dura and Vessels from Cortical Gray Matter

**DOI:** 10.3389/fnins.2017.00258

**Published:** 2017-05-09

**Authors:** Roberto Viviani, Eberhard D. Pracht, Daniel Brenner, Petra Beschoner, Julia C. Stingl, Tony Stöcker

**Affiliations:** ^1^Institute of Psychology, University of InnsbruckInnsbruck, Austria; ^2^Psychiatry and Psychotherapy Clinic III, University of UlmUlm, Germany; ^3^German Center for Neurodegenerative Diseases (DZNE)Bonn, Germany; ^4^Clinic for Psychosomatic Medicine and Psychotherapy, University of UlmUlm, Germany; ^5^Research Division, Federal Institute for Drugs and Medical DevicesBonn, Germany; ^6^Center for Translational Medicine, University of Bonn Medical SchoolBonn, Germany; ^7^Department of Physics and Astronomy, University of BonnBonn, Germany

**Keywords:** multimodal segmentation, cortex, dura, cortical vessels, voxel-based morphometry

## Abstract

While widely in use in automated segmentation approaches for the detection of group differences or of changes associated with continuous predictors in gray matter volume, T1-weighted images are known to represent dura and cortical vessels with signal intensities similar to those of gray matter. By considering multiple signal sources at once, multimodal segmentation approaches may be able to resolve these different tissue classes and address this potential confound. We explored here the simultaneous use of FLAIR and apparent transverse relaxation rates (a signal related to ${\text{T}}_{2}^{*}$ relaxation maps and having similar contrast) with T1-weighted images. Relative to T1-weighted images alone, multimodal segmentation had marked positive effects on 1. the separation of gray matter from dura, 2. the exclusion of vessels from the gray matter compartment, and 3. the contrast with extracerebral connective tissue. While obtainable together with the T1-weighted images without increasing scanning times, apparent transverse relaxation rates were less effective than added FLAIR images in providing the above mentioned advantages. FLAIR images also improved the detection of cortical matter in areas prone to susceptibility artifacts in standard MPRAGE T1-weighted images, while the addition of transverse relaxation maps exacerbated the effect of these artifacts on segmentation. Our results confirm that standard MPRAGE segmentation may overestimate gray matter volume by wrongly assigning vessels and dura to this compartment and show that multimodal approaches may greatly improve the specificity of cortical segmentation. Since multimodal segmentation is easily implemented, these benefits are immediately available to studies focusing on translational applications of structural imaging.

## Introduction

**Figure 1:**
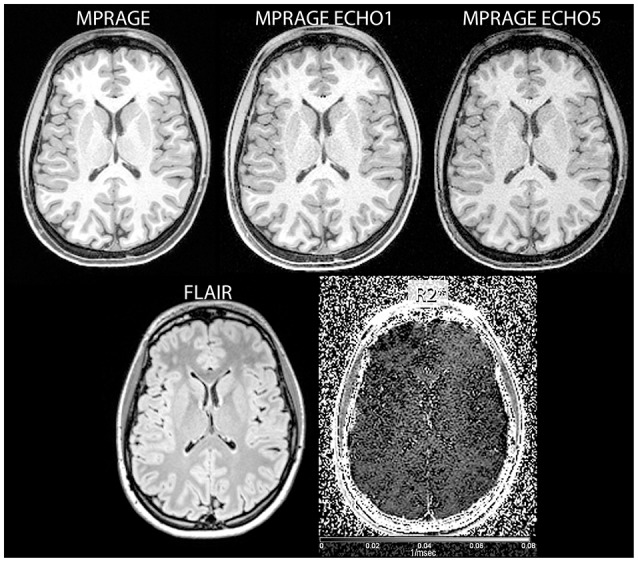
Examples of input images used in the study (clockwise from top left: reconstructed MPRAGE image, MPRAGE first echo, MPRAGE last echo, FLAIR, and ${\text{R}}_{2}^{*}$ images).

Probabilistic tissue classification methods represent one of the most important approaches to the investigation of brain structural differences *in vivo* with magnetic resonance imaging techniques (Zhang et al., 2001; Fischl et al., 2002, 2004; Ashburner and Friston, 2005). An important issue is the specificity of segmentation based, as commonly the case, on T1-weighted images. The use of multiple magnetic resonance images acquired with sequences with different contrast properties may improve the capacity of the segmentation process to resolve between tissue classes (“multichannel,” “multispectral,” or “multimodal” segmentation, Vannier et al., 1985; Fletcher et al., 1993; Alfano et al., 1997; Lambert et al., 2013a). Unlike work that increases contrast by non-linearly combining images acquired with different modalities into a single image (Misaki et al., 2015), multimodal segmentation considers multiple image types simultaneously and models the intensity of signal from tissue classes as a set of densities in multivariate space. This allows the algorithm, which summarizes the evidence for classification optimally given the density model, to identify the appropriate source of contrast to set the tissue classes apart. Here, we explored the use of multimodal segmentation to improve the accuracy of segmentation of cortical gray matter using combinations of MPRAGE, ${\text{R}}_{2}^{*}$, and FLAIR images (Figure 1).

**Figure 2:**
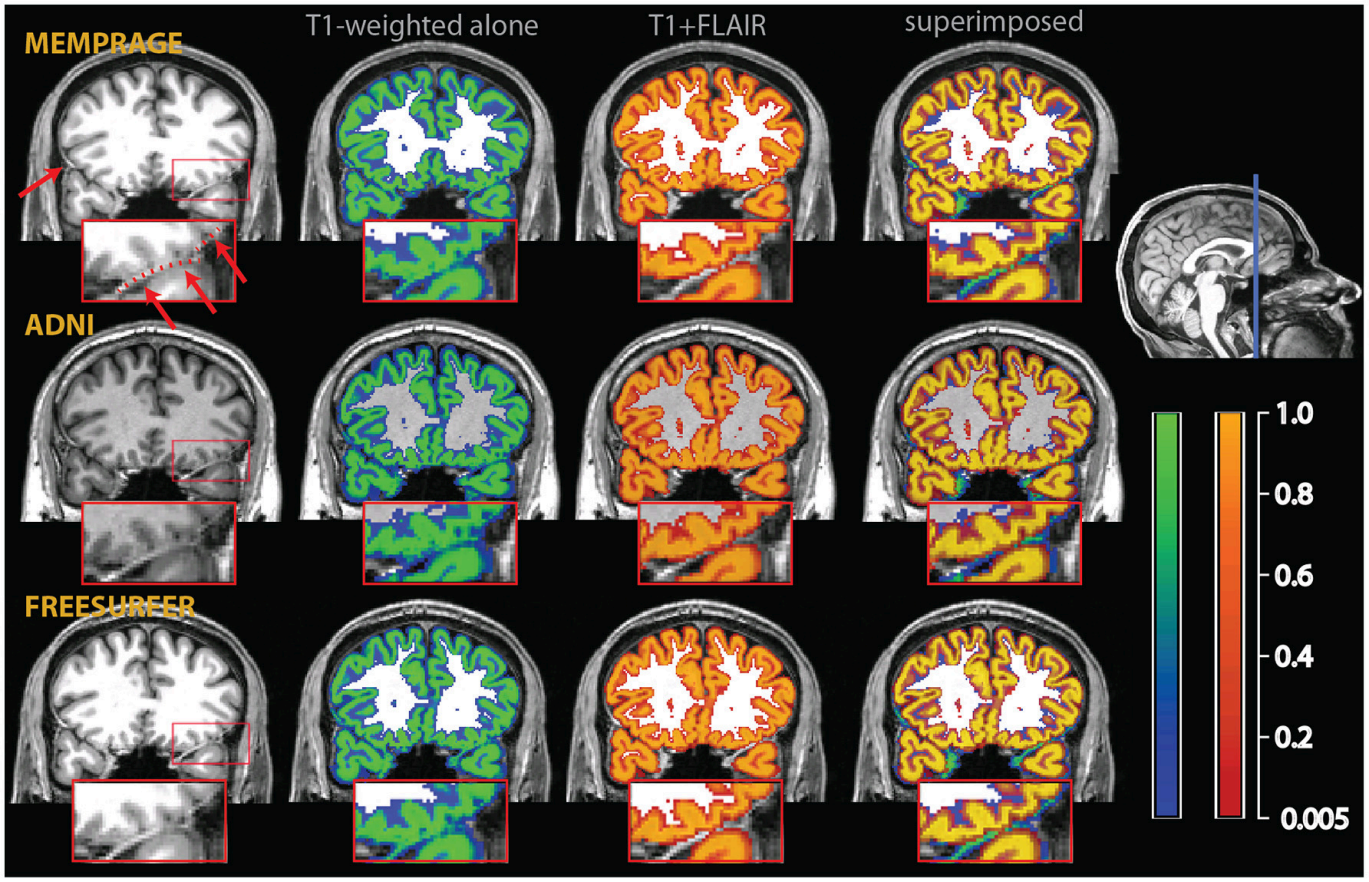
**Example of a misclassification of dura mater as gray matter**. The red arrows on the T1-weighted images on the upper left point to bright thick dura sections, highlighted by a dotted red line in the inset, which shows a magnified section of the affected area. In the central sections, tissue probability maps of the segmentation obtained from the T1-weighted images alone (overlay in blue-green) and the multimodal segmentation of the T1-weighted and FLAIR volumes (overlay in red-orange). On the right, the multimodal and the T1-weighted alone segmentations were superimposed to show the areas where the multimodal segmentation was more conservative in assigning voxels to the gray matter tissue class. There were no voxels in this image where the probability to belong to gray matter was substantially higher in the multimodal than in the t1-weighted alone segmentation.

One well-known problem in the segmentation of gray matter tissue, first highlighted by the community involved in the development and use of cortical thickness estimation methods, consists in the misclassification of dura mater as cortex (van der Kouwe et al., 2008). This problem is due to the equal intensity of the signal from dura and gray matter in many magnetic resonance sequences and is of variable severity across cortical regions and across individuals. In areas where the dura adheres to the cortex, it becomes difficult to distinguish it from gray matter, even when manual visual correction is attempted (see Figure 2).

**Figure 3:**
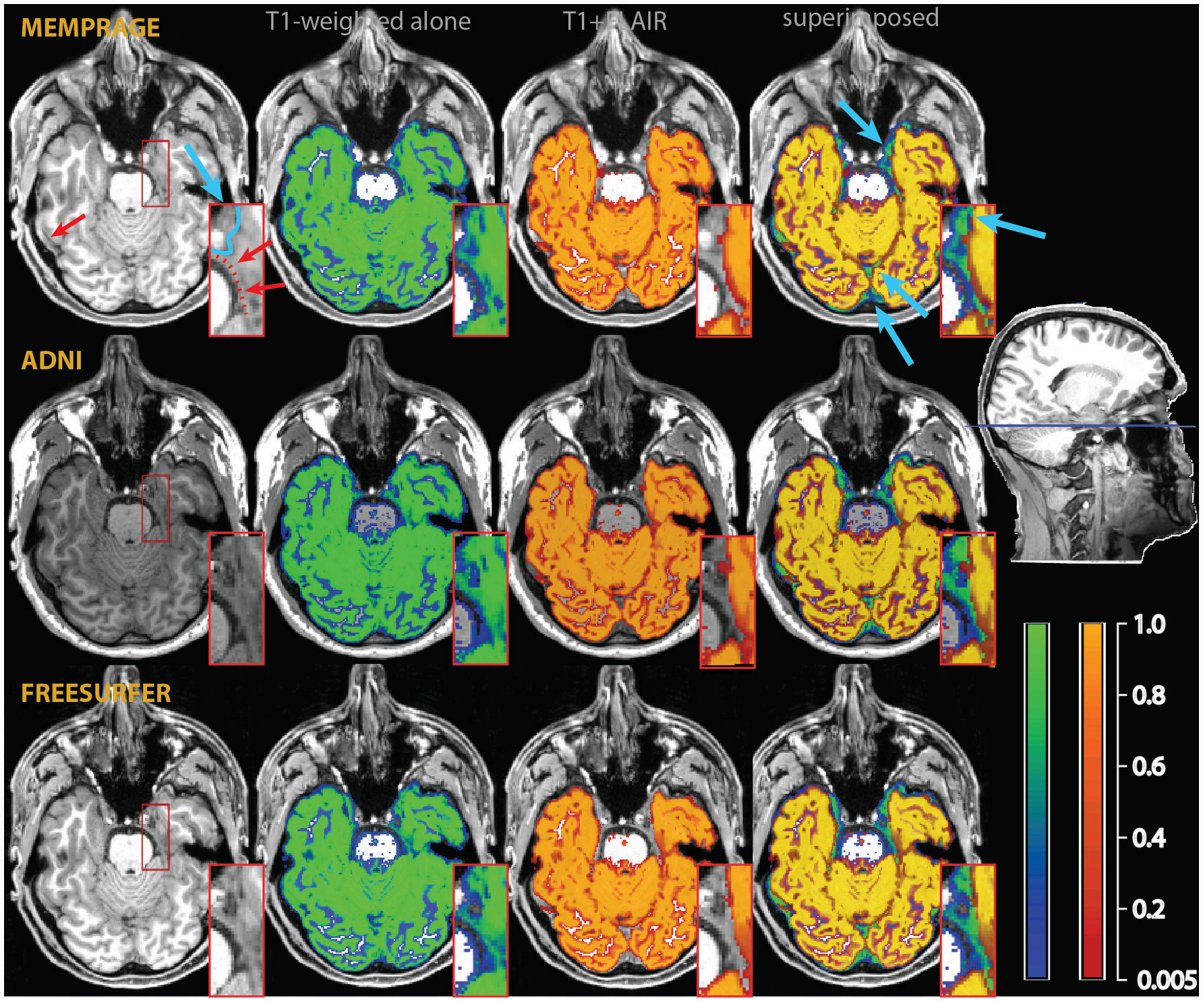
**Example of a misclassification of vessels as gray matter in the medial aspect of the temporal lobe (red arrows and dotted line in the inset in the top left)**. As in Figure 2, the segmentation of the T1-weighted images alone is shown as a blue-green overlay, and the multimodal segmentation with the added FLAIR images in red-orange. Light blue arrows point to differences in the classification of extracerebral tissue. The insets show an enlarged section of the medial right temporal lobe, where the improvements due to the multimodal segmentation are apparent.

**Figure 4:**
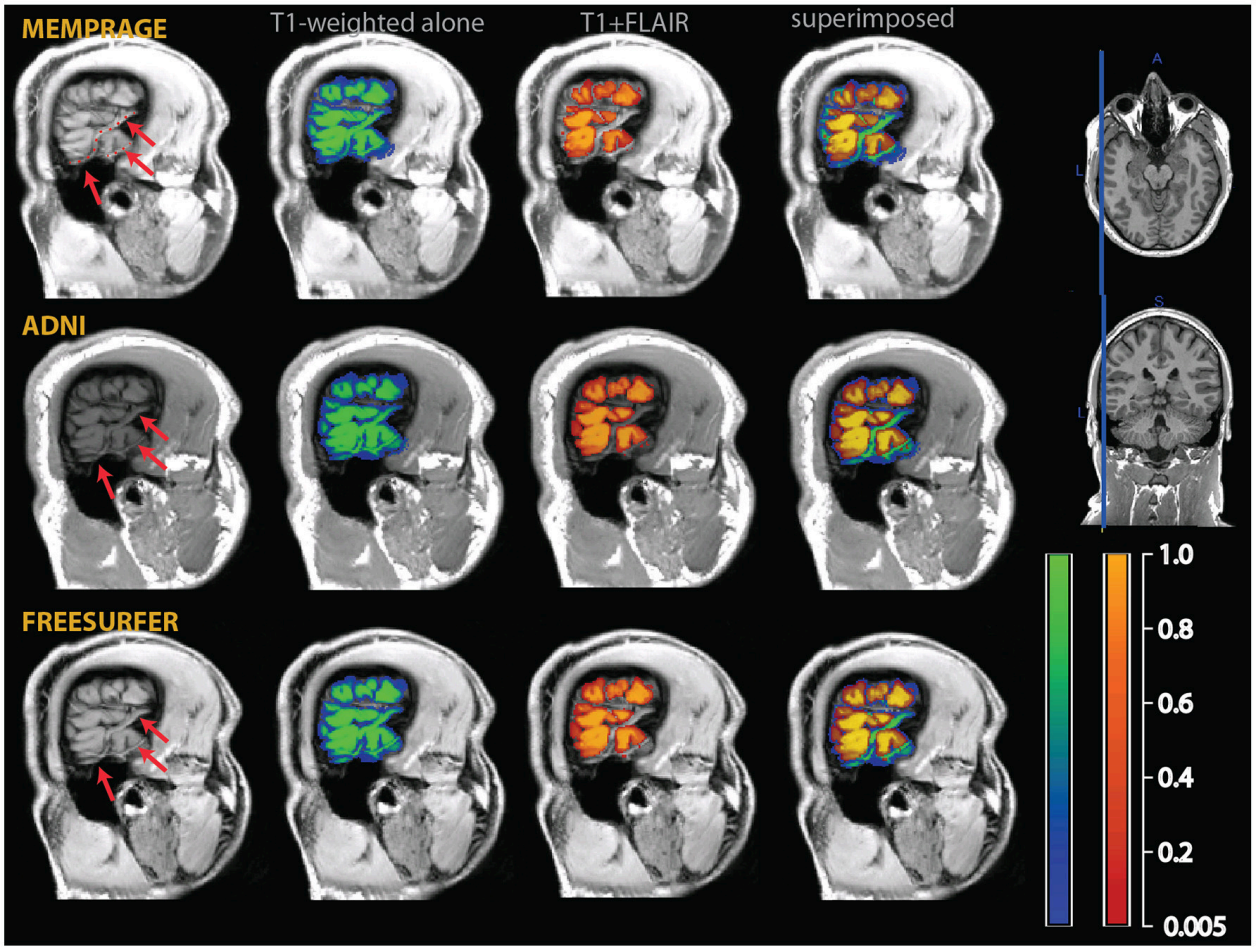
**Example of a misclassification of sulcal vessels as gray matter (red arrows and dotted red line in the T1-weighted images on the left)**. As in the previous figures, the segmentation of the T1-weighted images alone is shown as a blue-green overlay, and the multimodal segmentation with the added FLAIR images in red-orange. Note the vessels misclassified as gray matter by the T1-weighted segmentation (in green in the rightmost column of images), but not in the multimodal procedure.

A second, perhaps less widely known, problem is given by the misclassification of medium-size vessels as gray matter in common T1-weighted imaging protocols. This problem is potentially more severe in the areas where medium-sized vessels tend to congregate (Viviani, 2016), such as in the medial aspect of the cerebral hemispheres (due to the pericallosal arteries and their emissaries), in the deep folds of the Sylvian fissure, and on the medial and anterior face of the temporal lobes (mainly due to the middle and posterior cerebral arteries; Figure 3). More generally, medium-sized vessels running in sulci or in the vicinity of cortex may be occasionally misclassified as gray matter if giving rise to a bright signal as a consequence of inflow effects (Figure 4).

The purpose of the present study is the comparative evaluation of multiple signal sources in multimodal segmentation to address these possible misclassifications. While the benefits of using information from multiecho MPRAGE to resolve dura from gray matter tissue have been reported in the literature (van der Kouwe et al., 2008), little is known about the opportunities offered by multimodal segmentation to address the problem of the misclassification of vessels. Helms et al. (2006) could show that multimodal segmentation with T1- and T2-weighted images resolved signal from non-brain tissue such as draining sinuses and the adjacent connective tissue. However, no study has systematically addressed the comparative advantages of using different combinations of input images in multimodal segmentation approaches.

We considered two possible additional signal sources to the segmentation algorithm. In the first, we supplemented MPRAGE data with fluid-attenuated inversion recovery (FLAIR) images (Bydder and Young, 1985), and are increasingly used in segmentation and large imaging databases (Mendrik et al., 2015; Smith et al., 2017). Like appropriately weighted T2-weighted images, FLAIR images present differences in signal intensity between dura and cortical gray matter (Helms et al., 2006). Furthermore, these images also offer a good contrast between gray matter and cerebral fluid/white matter. In the second, we explored the utility of images of apparent transverse relaxation rates (${\text{R}}_{2}^{*}$ maps), combined with T1-weighted MPRAGE images or in a three-channel combination with MPRAGE and FLAIR. The apparent transverse relaxation rate ${\text{R}}_{2}^{*}$ is related to the effective relaxation time of the radiofrequency signal ${\text{T}}_{2}^{*}$ by the formula ${\text{T}}_{2}^{*}$ = 1/${\text{R}}_{2}^{*}$ (for a recent review, see Cohen-Adad, 2014). The use of relaxation estimates has historically represented the first approach to correct for the dura confound problem in the cortical thickness estimation literature (van der Kouwe et al., 2008). Our results show that the addition of either ${\text{R}}_{2}^{*}$ maps or FLAIR improves the specificity of the segmentation. However, in our study the MPRAGE+FLAIR combination emerged as clearly superior, albeit at the cost of increased overall acquisition times.

The rest of the paper is structured as follows. The Section Materials and Methods provides details on the sample, the MRI sequences used in the study, and the algorithms used in the analysis. In the Results Section we will examine different combination of input modalities in turn. We begin by providing illustrative examples of differences in the segmentation of cortical areas arising from the use of T1-weighted MPRAGE images alone and in the various combinations with FLAIR and ${\text{R}}_{2}^{*}$ maps. We continue by presenting the summary data of the whole sample, using the bi-modal and three-modal combinations of all signal types to visualize systematic differences in the segmentation due to the addition of the FLAIR and/or ${\text{R}}_{2}^{*}$ signals. We also provide a comparison with maps of vascular intensities predominantly showing the spatial distribution of arterial in-flow effects to identify where different segmentation outcomes may be attributed to a reclassification of this signal source. In the final part of the Results Section, we conclude with details on the estimates of the probability densities of the signal intensity in the brain tissue classes. These estimated densities, which are modeled as mixtures of Gaussians, give insight on how the algorithm “sees” the tissue classes and on the achieved separation between them.

## Materials and methods

### Sample and acquisition

The data were acquired using a Siemens 3T Prisma scanner located on the premises of the Clinic of Psychiatry and Psychotherapy III at the University of Ulm, Germany. This study was carried out in accordance with the recommendations of the Ethic Review Board of the University of Ulm. All subjects gave written informed consent in accordance with the Declaration of Helsinki. The protocol was approved by the Ethic Review Board of the University of Ulm. The data are part of a larger genetic neuroimaging database that is currently under acquisition. The sample consisted of 35 healthy participants (age mean/standard deviation 24.4/6.0, 18 females).

### MR acquisition

For our MPRAGE sequence we used a multi-echo MPRAGE sequence (MEMPRAGE) from which both the T1-weighted signal and ${\text{R}}_{2}^{*}$ maps were obtained. The MEMPRAGE sequence had a high readout bandwidth, matched to the readout bandwidth of the FLAIR sequence. The high bandwidth minimizes differential distortions in the two sequences, and offers the theoretical advantage of reducing the effects of susceptibility artifacts in the gradient-echo MPRAGE images. Besides obtaining an ${\text{R}}_{2}^{*}$ star signal without requiring additional scanning time, matching the readout bandwidth was an important motivation in the adoption of this sequence. Relative to the MPRAGE+FLAIR combination, the MPRAGE+${\text{R}}_{2}^{*}$ maps approach has the advantage that only the MEMPRAGE imaging session is required. MEMPRAGE images were acquired with isotropic voxel size 1 mm, FOV 256 × 256, 176 sagittal slices, phase encoding anterior-posterior, TR = 2,500 ms, TE = 1.48, 2.98, 4.48, 5.98, 7.48 ms, TI = 1120 ms, bandwidth 780 Hz/pixel, non-selective inversion recovery, flip angle 7°, acquisition time 4 min 43 s. The MPRAGE image was obtained by averaging the images obtained at all echo times.

FLAIR images were acquired with isotropic voxel size 1 mm, FOV 256 × 256, 176 sagittal slices, phase encoding anterior-posterior, TR = 5,000 ms, TE = 397 ms, TI = 1,800 ms, bandwidth 781 Hz/pixel, non-selective inversion recovery, flip angle 120°, acquisition time 4 min 42 s. In two participants two additional MPRAGE images were acquired using standard protocols (“Freesurfer” and “ADNI”). The acquisition parameters for these protocols were obtained online from https://surfer.nmr.mgh.harvard.edu/fswiki/ and from http://adni.loni.usc.edu/methods/documents/mri-protocols/. Data from these two participants were used to draw Figures 2–4, in which the results the simple MPRAGE and combined segmentations may be compared. The acquisition parameters and the processing stages of the time-of-flight images of Figure 6 are described in Viviani (2016).

### Algorithms and software used

To obtain the apparent relaxation rate maps (${\text{R}}_{2}^{*}$ maps) in each individual the formula described in Hagberg et al. (2002) was used. Due to the larger variability of estimated ${\text{T}}_{2}^{*}$-values, these maps provided better segmentation results than images based on the estimated ${\text{T}}_{2}^{*}$ times, either when used alone or in combination with the T1-weighted data.

All further processing was carried out with the freely available SPM software package (version 12, Welcome Trust Centre for Neuroimaging, University College London, http://www.fil.ion.ucl.ac.uk/spm/). FLAIR images were preliminarily co-registered to the MEMPRAGE T1-weighted images. MPRAGE, apparent relaxation rates-, and FLAIR images were included (as indicated by the combination at hand) in the “unified segmentation” procedure available in this software package (Ashburner and Friston, 2005). Figures 2–4 display gray matter probability maps at the original voxel size 1 mm. All other Figures refer to “modulated” probability maps for the analysis of volumetric effects- resampled at voxel size 1.5 mm. Bhattacharyya distance, a measure of overlap between two densities, is given in the multivariate case by the formula $\frac{1}{8}(\mu_{1}-\mu_{2})\prime \Sigma^{-1}(\mu_{1}-\mu_{2})+\frac{1}{2}\ln\left(\left|\sum \right|/\sqrt{\left|\sum_{1}\right|\left|\sum_{2}\right|}\right)$, where μ_*i*_ and Σ_*i*_ are the multivariate means and covariances, $\sum =\frac{1}{2}(\sum_{1}+\sum_{2})$ and |·| denotes the determinant (Kailath, 1967). All computations were carried out with the software package MATLAB, version 12. Overlay images were produced with the freely available software MRIcron (Chris Rorden, http://www.mccauslandcenter.sc.edu/mricro).

## Results

### Segmentation MPRAGE+FLAIR

The results of the segmentation were assessed in two ways. The first consisted of visualizing tissue probability maps individually to compare the outcome of the segmentations based on the MPRAGE images alone and on the MPRAGE+FLAIR combination. The second consisted of the generation of parametric maps of differences of mean segmentation outputs to visualize systematic differences between the segmentations.

In the individual assessment, the MPRAGE+FLAIR combination presented very good results, with no instances where visual inspection detected errors in the classification of vessels or dura. The addition of the FLAIR signal resulted in a more conservative assignment of voxels to the gray matter tissue class with the exception of a few specific areas (detailed below). This occurred because the main sources of different classification in the multimodal procedure, dura and vessels, both tended to decrease the number of voxels assigned to gray matter. The addition of the FLAIR input modality made little difference to the classification of the gray matter tissue class in most voxels, except for the three conditions described above, i.e., dura, vessels, or connective tissue at the edge of the brain. The latter were particularly pronounced at the swaths of tissue located between the occipital and calcarine cortex and the cerebellum. Examples of such individual comparisons for two participants are shown in Figures 2–4.

To assess the extent to which the misclassification may be due to the specific parameters of our acquisition protocol, we also acquired T1-weighted images using standard MPRAGE acquisition protocols in the two participants of Figures 2–4 (in the rest of the sample, only the MEMPRAGE images were acquired). The misclassification (when T1-weighted images were used alone) in the segmentation was similar irrespective of the MPRAGE protocol used and the changes following the inclusion of the FLAIR signal were almost identical.

In Figure 2, one can see that the misclassified dura was assigned a probability of ~0.8 to belong to gray matter in the segmentations using the T1-weighted images alone, but a probability of about zero in the multimodal segmentations. Similarly, in Figures 3, 4, which show misclassification of vessels in the medial aspect of the temporal lobe and in the cortical sulci, the T1-weighted segmentations classified the vessels as gray matter with a probability about ~0.8 where the multimodal segmentations set this probability to about zero.

Figure 3 also illustrates how conservative the T1-weighted+FLAIR combination was in assigning voxels to gray matter at the edge of the brain in places where gray matter was adjacent to connective tissue. One example is the anterior pole of the right temporal lobe (also shown in the inset). Here, connective tissue surrounded vascular tissue and the optic nerve. A similar effect of the addition of the FLAIR signal is apparent in the occipital pole/calcarine cortex, at the border with cerebellar tissue (light blue arrows). Here, large swaths of connective tissue that is isointense to gray matter in the T1-weighted signal may be classified as non-brain when the T2-weighted FLAIR signal is available (Helms et al., 2006).

**Figure 5:**
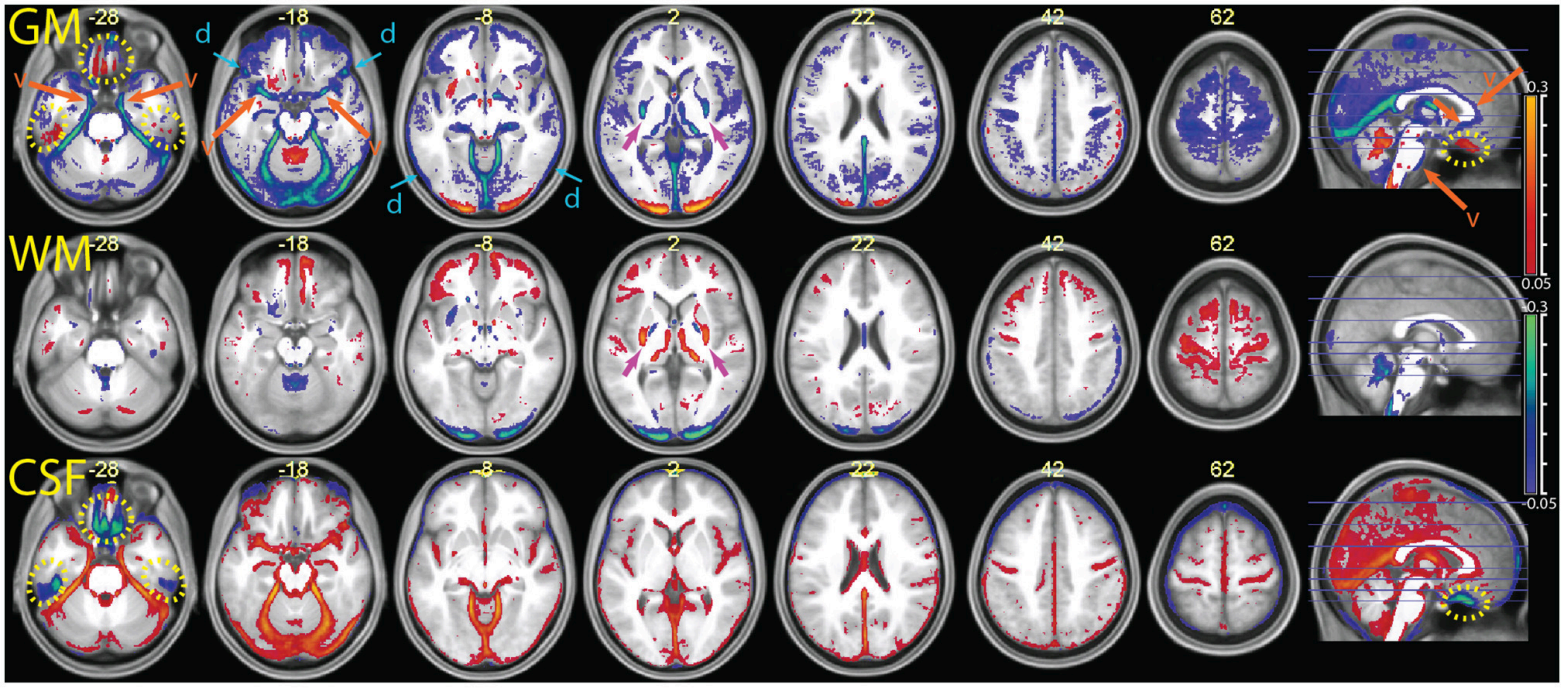
**Comparison of the segmentation obtained with the multimodal procedure using MPRAGE and FLAIR images, relative to a segmentation obtained with the MPRAGE images alone (difference of averaged tissue probability maps)**. In blue, values that decrease in the multimodal procedure; in red, values that increase. From top to bottom the segmentation output values for gray matter (GM), white matter (WM) and cerebrospinal fluid (CSF) were overlayed on the mean normalized MPRAGE image (coordinates in mm, MNI space). The yellow dotted circles highlight areas affected by susceptibility artifacts (visible at *z* –28 and in the sagittal images). The orange arrows and letter “v” point to areas where the multimodal segmentation gave different results due to detection of vessels (visible at *z* –28, –18 and in the sagittal image). The blue arrows at *z* –18 and –8 show decreases in the GM values in areas affected by the dura problem (present also in slices above). One can also see a shift in intensity from the GM to the WM compartment in the putamen, especially in its posterior portion (magenta arrows at z 2). Decreases of estimated GM volume amounted to an average 67 ± 21 ml in the combined relative to the simple segmentation. In the combined segmentation, WM and CSF volumes were on average 12 ± 8 and 25 ± 35 ml larger, respectively.

The parametric maps of the difference between the average tissue probability maps in the MPRAGE+FLAIR combination and the segmentation with MPRAGE alone (Figure 5) summarize these differences for the entire sample (in this and the following figures, we refer to “CSF” for the tissue class that includes the ventricles and liquor-filled spaces, although this is in reality a somewhat heterogeneous compartment whose precise content changes according to the combination of inputs to the segmentation algorithm). The data are put in register through the “unified segmentation-normalization” procedure that is integrated in the segmentation algorithm. One can see that the predominant change in the multimodal MPRAGE+FLAIR combination was a decrease in the areas assigned to the gray matter compartment (in blue-green in the top row of the figure; the few exceptions in which assignment to gray matter increased are discussed below). This is consistent with the different assignment of voxels representing dura of vessels noted in the individual analysis of Figures 2–4. Figure 5 also reveals that shifts in the classification away from gray matter were particularly apparent in lower slices (where the misclassification of the dura may be expected to be more prominent, van der Kouwe et al., 2008; Ribes et al., 2011), and in correspondence of large and medium-sized vessels.

**Figure 6:**
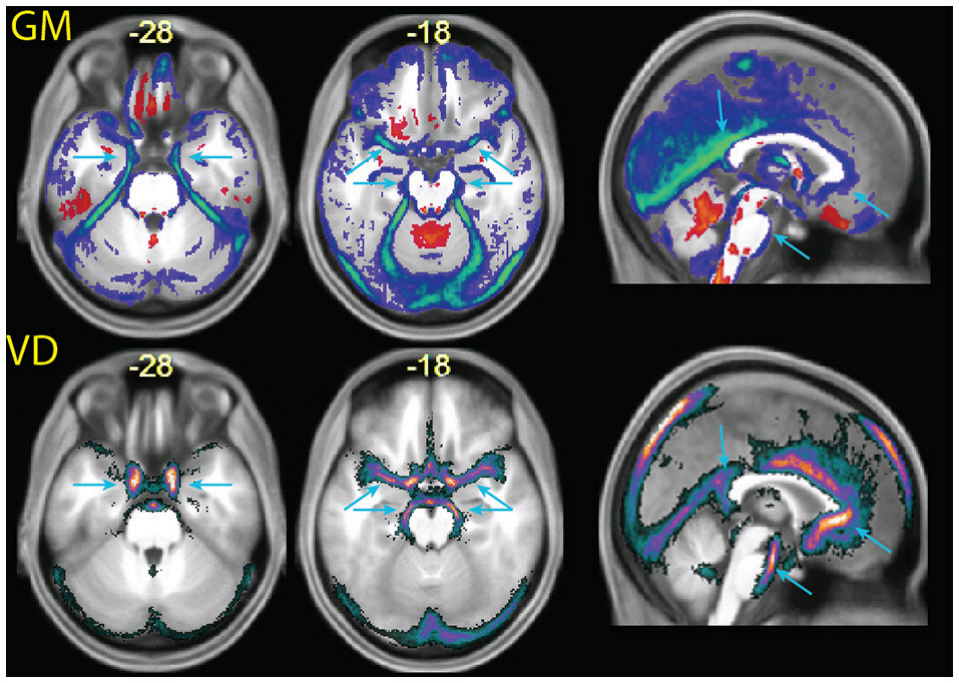
**(Top row)** enlarged slices from Figure 5, showing differences in the tissue probability maps for gray matter (GM) obtained with the MPRAGE+FLAIR combination relative to the MPRAGE segmentation. **(Bottom row)** images of vascular density (VD), obtained from a publicly available vascular density atlas from time-of-flight images segmented semimanually (Viviani, 2016). The top of the brain is not represented in these data due to missing coverage. Note that the multimodal segmentation identifies more tissue as non-gray matter than the time-of-flight based data (for example around the cerebellum in the center slice), due to the different intensity of connective tissue in the T2-weighted signal (Helms et al., 2006).

To validate the vascular origin of these classification differences, we compared them to images of vessel frequency obtained from time-of-flight images (Figure 6). The figure shows that some of the decreases in gray matter probability maps in the combined MPRAGE + FLAIR segmentation were due to vascular signal (blue arrows). This was especially the case at the anteromedial border of the temporal lobe (transversal slices). Vessel signals may also have contaminated T1-weighted estimates of gray matter tissue at the border of the anterior half of the corpus callosum and the pons.

Figure 6 also shows extensive areas of reduced gray matter in the MPRAGE+FLAIR segmentation corresponding to the cerebellar tentorium, around the cerebellum, especially at the junction with the inferior occipital lobes and in the medial aspect of the anterior temporal pole. These areas correspond to tissue with different intensity in the time-of-flight images due to the presence of large collectors of venous blood (the time-of-flight images were acquired without the saturation band at the top of the head that suppresses venous signals in clinical applications of this sequence). The same areas changed in the individual of Figure 3 (light blue arrows), where the T1-weighted signal gave no indication regarding the extracortical nature of this tissue. Changes in gray matter posterior to the splenium of the corpus callosum corresponded to the location of the great cerebral vein. These changes in the classification of tissue adjacent to cortical gray matter attributable to large venous collectors and the surrounding connective tissue are similar to those reported by Helms et al. (2006) in their investigation of the T1- and T2-weighted combined segmentation.

Two notable exceptions to the general pattern of reduced gray matter in the MPRAGE+FLAIR segmentation were given by the medial orbitofrontal cortex and the concavity of the inferior aspect of the temporal lobes (yellow dotted ovals in Figure 5). Being adjacent to the sphenoid sinus and mastoid air cells, these areas are typically affected by magnetic susceptibility-induced artifacts. This led to signal dephasing and signal loss, which were more marked in the gradient-echo T1-weighted MPRAGE images than in the spin-echo FLAIR images. A comparison with the values assigned by the segmentation to the CSF compartment (bottom row) shows that in these areas the combined MPRAGE+FLAIR segmentation recovered signal from gray matter assigned to CSF in the MPRAGE segmentation alone.

**Figure 7:**
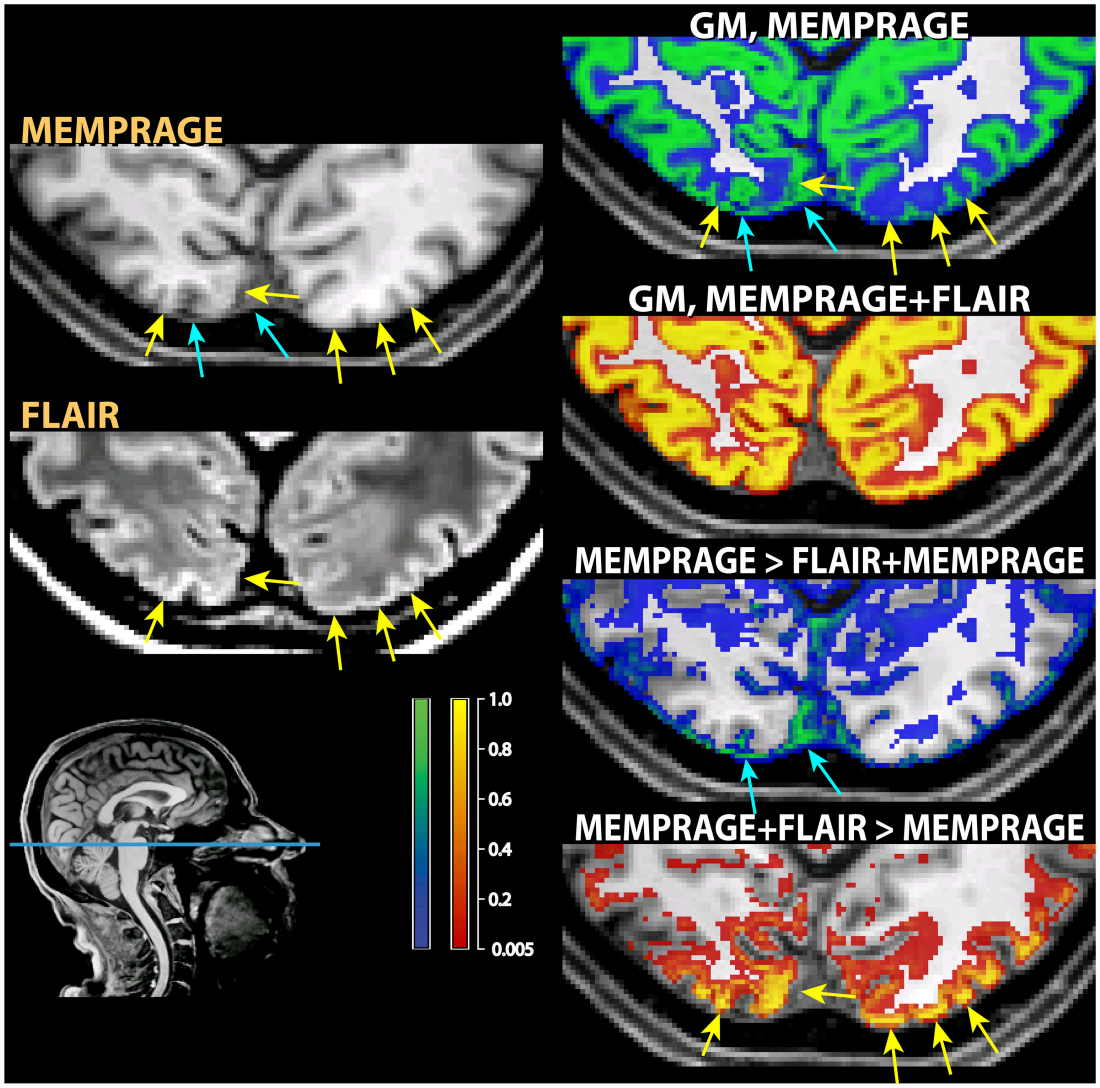
**Example of classification differences in the occipital cortex**. As in those figures, the segmentation of the T1-weighted images alone is shown as a blue-green overlay, and the multimodal segmentation with the added FLAIR images in red-orange. In the left column the input MPRAGE and FLAIR image are shown. The yellow arrows show loss of contrast in the T1-weighted MPRAGE images, which was retained in the FLAIR images. The right column show the classification outcome in the MPRAGE alone segmentation (top, in green) and in the MPRAGE+FLAIR combination (in red-yellow). The yellow arrows pointing to loss of contrast show in the MPRAGE alone segmentation that these areas achieved a classification of about 0.1–0.2 as gray matter, but 0.9 or more in the combined MPRAGE+FLAIR combination. The images in the bottom right corner show the difference in the classification outcome. Note that the parts adjacent to the cortex where the MPRAGE alone segmentation attributed a higher classification probability than the MPRAGE+FLAIR combination (blue arrows) were not gray matter, but connective tissue. This is apparent from the inspection of the input MPRAGE image, where the dura is evident as a continuous sheet running over the left half of the figure, isointense to gray matter in the MPRAGE data but giving low signal in the FLAIR image.

A further exception to the general pattern was the increased gray matter in the occipital cortex of the posterior pole of the brain and, to a much smaller extent, in the rectus and orbital gyri in the orbitofrontal cortex. Comparison with the values assigned to white matter (middle row) shows that the increased gray matter values occurred at the expenses of the white matter compartment. This stood in contrast to the general effect of the addition of the FLAIR signal, which tended to shift the classification of voxels at the gray-white matter boundary toward white matter. The visual inspection of individual MPRAGE images revealed that the white/gray matter contrast of the T1-weighted signal in the occipital cortex was very low, compounding possible partial volume effects from white matter arising from the thinness of the cortical ribbon (Figure 7). The low contrast is explained by the high cortical myelin content of this part of the cortex (Walters et al., 2003; Eickhoff et al., 2005), as identified in appropriately configured segmentations that include the T2-weighted signal of FLAIR (Viviani et al., 2017).

There were further differences in the outcome of the segmentation in the brainstem and in the basal ganglia. In the latter, we observed a decrease of the gray matter values in the combined MPRAGE+FLAIR segmentation in the posterior putamen, which was shifted toward white matter (visible at *z* = 2 in Figure 5). Less marked, similar shifts were observed in the anterior pallidum and the substantia nigra. These differences are consistent with the sensitivity of gradient-echo images to differences in tissue iron content (Drayer et al., 1986; Haacke et al., 2005; Pfefferbaum et al., 2009). Elsewhere, decreased gray matter values may have been due to an increase in contrast with the white matter of the internal capsule. The addition of the FLAIR images also appear to have captured signal intensity differences in the thalamic laminae (also visible in the slice at *z* = 2; Figure 5).

### Segmentation MPRAGE+${\text{R}}_{2}^{*}$

**Figure 8:**
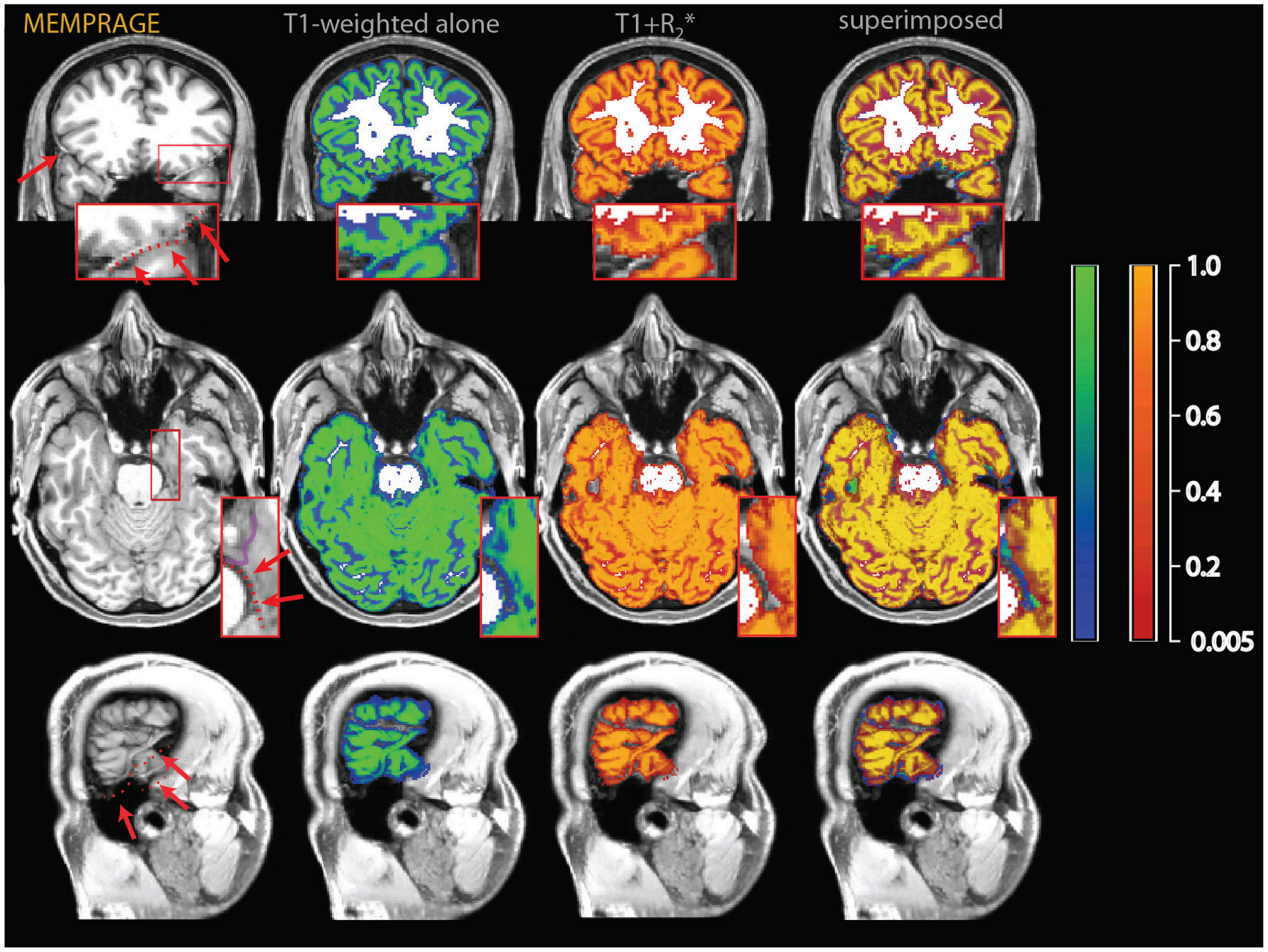
Data from the two individuals of Figures 2–4, here comparing the outcome of the segmentation of the T1-weighted data alone (identical to the data of Figures 2–4) and the outcome of the combination T1-weighted+${\text{R}}_{2}^{*}$.

**Figure 9:**
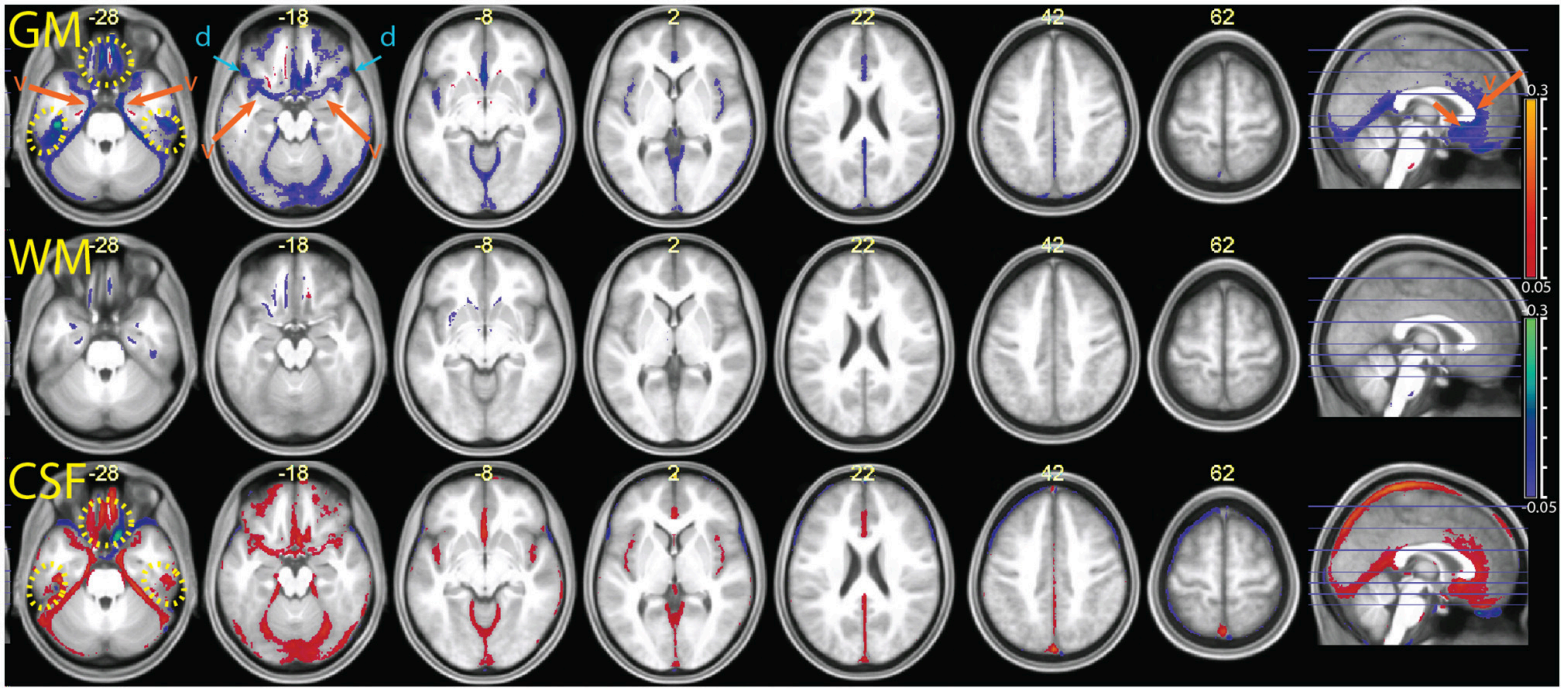
Comparison of the segmentation obtained with the multimodal procedure using MPRAGE images and the ${\text{R}}_{2}^{*}$ signal, relative to a segmentation obtained with the MPRAGE images alone. Decreases of estimated GM volume amounted to an average 26 ± 11 ml in the combined relative to the simple segmentation. There were no appreciable differences in the WM volumes in the combined and simple segmentation (<1 ml). In the combined segmentation, CSF volumes were on average 11 ± 20 ml larger than in the simple segmentation. Colors and symbols as in Figure 5.

The comparison between the MPRAGE+${\text{R}}_{2}^{*}$ (apparent transverse relaxation rates) and the MPRAGE segmentations is shown in Figures 8, 9. Some aspects of the different classification in the multimodal procedure are similar to those visible in the MPRAGE+FLAIR combination, but the degree of correction at the outer surface of the cortex, where we would expect the dura problem to be most prominent, was smaller. Visual inspection revealed that the addition of the ${\text{R}}_{2}^{*}$ maps led to a reduction of the tissue probability map values assigned to gray matter in the voxels affected by the misclassification (see top row of Figure 8). This effect, particularly apparent in the lower slices, is reflected in the lower average gray matter values at the edge of the brain (visible in Figure 9). In large vessels, the MPRAGE+${\text{R}}_{2}^{*}$ tissue probability maps showed large decrements in the gray matter values, similarly to the MPRAGE+FLAIR combination (middle row of Figure 8). However, the MPRAGE+FLAIR combination was more effective in detecting medium-sized vessels in the cortex (compare the bottom row of Figures 4, 8). This is reflected in the diffuse shift toward CSF of the cortical gray matter probability maps of the MPRAGE+FLAIR segmentation of Figure 5, which was absent in the MPRAGE+${\text{R}}_{2}^{*}$ data of Figure 9.

In the areas of the orbitofrontal and temporal cortex where there was an increase of the gray matter tissue probability map values in the MPRAGE+FLAIR combination, the opposite effect was observed here (yellow dotted circles in Figure 9). This is consistent with increased magnetic susceptibility-induced artifacts. At their peaks, the average gray matter value reductions in these areas in the combined MPRAGE+${\text{R}}_{2}^{*}$ relative to MPRAGE segmentations were considerable, amounting to over 30%.

### Segmentation MPRAGE+${\text{R}}_{2}^{*}+$FLAIR

**Figure 10:**
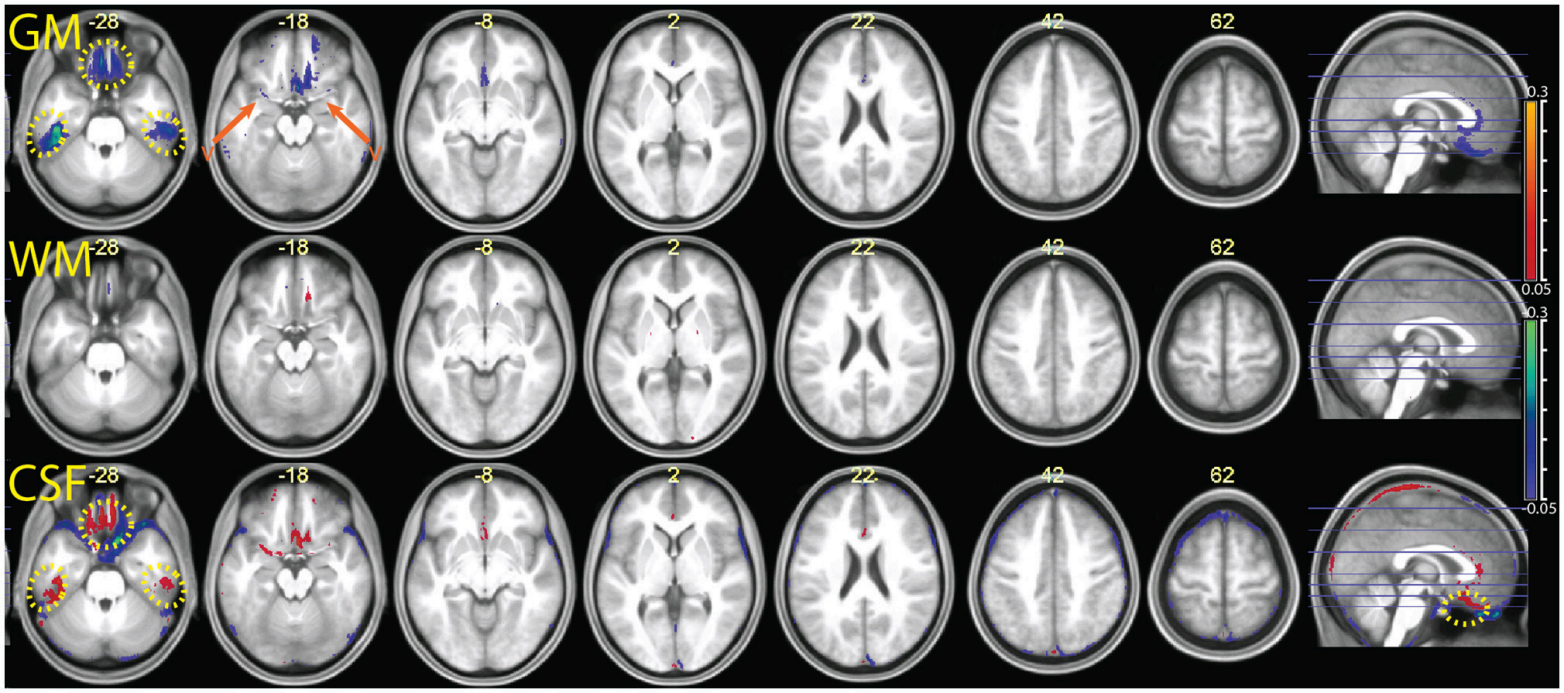
Comparison of the segmentation obtained with the multimodal procedure using MPRAGE images, FLAIR images, and the ${\text{R}}_{2}^{*}$ maps, relative to the segmentation obtained with the combination MPRAGE + FLAIR images. Decreases of estimated GM volume amounted to an average 9 ± 9 ml in the MPRAGE+FLAIR+${\text{R}}_{2}^{*}$ relative to MPRAGE+FLAIR. There were no appreciable differences in the WM volumes in the combined and simple segmentation (<1 ml). Effects on the CSF volume were not consistent. In the MPRAGE+FLAIR+${\text{R}}_{2}^{*}$ segmentation, CSF volumes increased on average by 14 ± 28 ml than in the MPRAGE+FLAIR segmentation. Colors and symbols as in Figure 5.

In Figure 10 we show the comparison between the multimodal segmentation using all sources of signal (MPRAGE, ${\text{R}}_{2}^{*}$ maps, and FLAIR) and the combination MPRAGE+FLAIR. Because of the good performance of the MPRAGE+FLAIR combination, our intent was to investigate if the addition of ${\text{R}}_{2}^{*}$ maps would add any useful information to the MPRAGE+FLAIR combination.

The inspection of Figure 10 reveals that the addition of ${\text{R}}_{2}^{*}$ maps to FLAIR only led to minor differences in the output of the segmentation procedure. Possible exceptions here were small improvements in the detection of vessels. One also sees that in the orbitofrontal cortex and in the inferior temporal lobes the use of the ${\text{R}}_{2}^{*}$ maps led to decreases in signal intensity for gray matter relative to the output of the MPRAGE+FLAIR segmentation, as we would expect from the sensitivity of the apparent transverse relaxation signal to magnetic susceptibility-induced artifacts.

### Tissue class density separation in the tissue class density models

**Figure 11:**
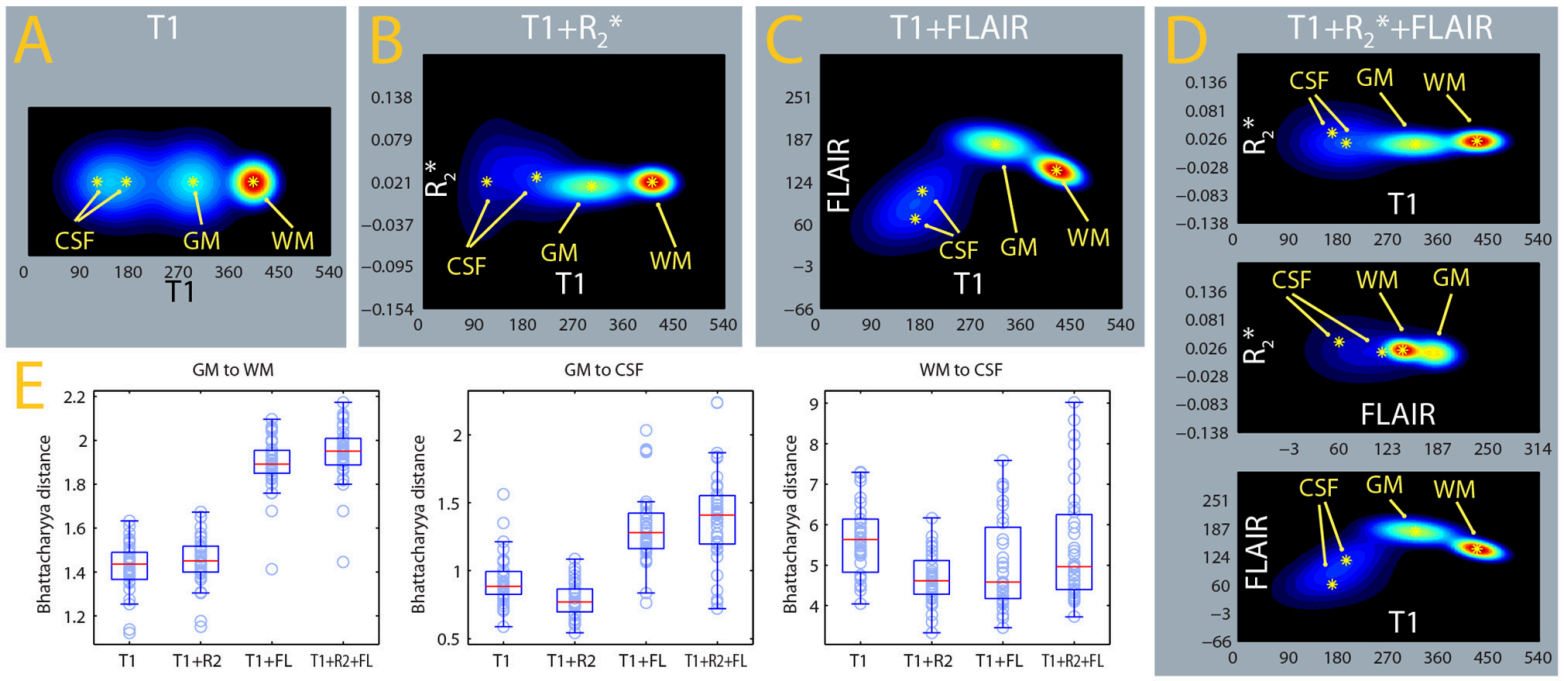
**Estimates of tissue class densities and relative separation. (A–D)** : average signal densities from the MPRAGE, MPRAGE+${\text{R}}_{2}^{*}$, MPRAGE+FLAIR, and MPRAGE+${\text{R}}_{2}^{*}+$FLAIR segmentations. The estimate from the MPRAGE segmentation alone was displayed as a two-dimensional density to facilitate comparison. The gray matter (GM) and white matter (WM) tissue classes were modeled with one Gaussian, while cerebrospinal fluid (CSF) was modeled as a mixture of 2 Gaussians. **(E)** Box-plots of the Bhattacharyya distances between the tissue classes computed in the individual segmentations. The distance of GM to CSF (box-plot in the middle) was computed to the Gaussian with largest weight in the mixture.

The algorithm used here classifies voxels in tissue classes according to their signal intensities, which are evaluated in relation to estimated distributions of this signal for each tissue class (Ashburner and Friston, 2005). In Figures 11A–D, we show the estimated densities for the gray matter, white matter, and CSF tissue classes, averaged from the whole sample. A mixture of two Gaussians was used to model the signal intensity of CSF and one Gaussian each for gray and white matter, as in usual applications of this software package to the segmentation of gray matter from T1-weighted images. One can see that the separation between the gray matter and CSF densities is considerably better in the MPRAGE+FLAIR than in the MPRAGE+${\text{R}}_{2}^{*}$ combination and in the MPRAGE alone case. The larger separation of the densities is consistent with the improvements in the identification of dura and vessels, which depends on the densities estimated for gray matter and CSF. This visual impression is confirmed by the comparison of the Bhattacharyya distances between the Gaussians, which quantify the distance between the centers of mass of the densities while taking their covariances into account (Figure 11E, GM to CSF, Table 1). As one can see from Figure 11E and Table 1, the MPRAGE+FLAIR model achieved a better separation of gray matter from CSF than the MPRAGE+${\text{R}}_{2}^{*}$ combination or MPRAGE alone (the increased distances are statistically significant at *p* < 0.001, paired sample *t*-test). The increased GM-CSF distances of the centers of mass of the MPRAGE+${\text{R}}_{2}^{*}+$FLAIR relative to the MPRAGE+FLAIR combination, in contrast, were not significant due to the high variance in these data.

**Table 1:** **Comparative summary of the Bhattacharyya distances of the Gaussian components modeling tissue intensity distributions estimated in different segmentations**.

**Component**	**Weight**	**Bhattacharyya distance**		
		**WM**	**CSF1**	**CSF2**
**T1**				
GM	1	1.55	0.91	2.15
WM	1		5.60	10.37
CSF1	0.58			0.20
CSF2	0.42			
**T1 + R**${\text{R}}_{2}^{*}$				
GM	1	1.44	0.77	3.25
WM	1		4.51	14.36
CSF1	0.61			0.67
CSF2	0.39			
**T1 + FLAIR**				
GM	1	1.87	1.27	3.65
WM	1		4.64	5.23
CSF1	0.68			0.22
CSF2	0.32			
**T1 + R**${\text{R}}_{2}^{*}$+ FLAIR				
GM	1	1.92	1.34	3.68
WM	1		4.95	5.29
CSF1	0.69			0.37
CSF2	0.31			

Figure 11E and Table 1 also show that the addition of the FLAIR signal in the segmentation increased the separation of the densities of gray and white matter (GM to WM). This is consistent with the observation that the MPRAGE+FLAIR segmentation was much better in estimating the cortical mantle at the occipital pole (Figure 5). The average distance between the centers of white matter and CSF (WM to CSF) may vary without practical consequences, because these two densities have no overlap.

## Discussion

The addition of an input modality to the segmentation procedure was predominantly accompanied by improvements of the specificity of the classification of voxel in the gray matter compartment, as confirmed by visual inspection of the causes of the differences observed in the aggregate images. As anticipated, the improvements occurred because of the increased identifiability of the dura and vessel signal disturbances but also of the better classification of connective tissue and venous collectors at the edge of the brain. The improvements were particularly marked in structures were documenting atrophy is of key importance, such as the medial and inferior face of the hippocampus. However, these improvements were much larger when the FLAIR signal, rather than the apparent relaxation rate ${\text{R}}_{2}^{*}$, was added in the multimodal segmentation. One may conclude that the addition of the signal from the multiple echos to the segmentation procedure may improve the quality of the outcome only when FLAIR data are not available. The addition of the FLAIR signal also improved the detection of the thin cortical mantle in the occipital poles. The FLAIR images also corrected small but consistent drops in signal intensity for the gray matter compartment in areas affected by susceptibility artifacts in the data from T1-weighted images alone, which is consistent with the proneness of gradient echo techniques to these artifacts (Reichenbach et al., 2005).

A limitation of the present study with respect to the estimation of the ${\text{R}}_{2}^{*}$ maps consisted of the short echo intervals of the MEMPRAGE sequence, whose rationale was to evaluate the added value of this signal with no additional acquisitions. ${\text{R}}_{2}^{*}$ maps may be measured with improved precision at longer echo times. However, even at the short echo times of our study, we observed an increase of susceptibility artifacts in the segmentation that included ${\text{R}}_{2}^{*}$ maps, suggesting that data from longer echo times would be prone to even larger dephasing effects.

An interesting effect of the addition of the FLAIR signal to the multimodal segmentation procedure was given by differences in the segmentation output in the basal ganglia and the brainstem. These differences are consistent with the different contrast properties of MRI protocols. The effect of iron on T2-weighted images, in particular, may have been felt in the segmentation of subcortical structures, compounding problems in the correct attribution to gray matter that affect segmentations of MPRAGE images (Lorio et al., 2016). These changes show that the addition of new input modalities in a segmentation procedure may introduce or strengthen sources of contrast that do not necessarily reflect volumetric differences.

It is important to draw attention to the significance and benefits of multimodal segmentation as such that emerge in the present study. First, if the signal has good signal-to-noise properties, inclusion of multiple input modalities can only increase the specificity of segmentation due to the additional sources of contrast. The present study shows that, in the case of cortical gray matter, the gains may be substantial. Second, multimodal segmentation is not only feasible but also easy to implement. Given the current interest in using structural imaging in characterizing disorder subgroups or predicting their course, the increased specificity of multimodal segmentation is immediately available in studies of key translational significance. Third, to date little has been done to explore the opportunities offered by multimodal segmentation. Beside its utility in differentiating cortex from dura and vessels, it may also be useful in identifying subcortical gray matter (Derakhshan et al., 2010; Datta and Narayana, 2013), brainstem structures (Lambert et al., 2013a,b), cerebral lesions (Alfano et al., 2000; Van Leemput et al., 2001; Engström et al., 2014) and, for what concerns cortical gray matter, identifying morphological differences in cortical myelin content (Viviani et al., 2017). To fully realize the potential of multimodal segmentation, it will be important to explore models of signal density in which the number of Gaussian components is varied to identify additional features of tissue (Viviani et al., 2017). This suggests that, in addition to increased specificity, multimodal segmentation alone or in combination with quantitative techniques may expand the amount of information that we may gain noninvasively with MRI.

## Author contributions

Designed research: RV, JS, EP, and TS. Contributed sequences: EP and DB. Collected data: EP and PB. Analyzed data: RV, PB, and EP. Interpreted data: RV, EP, JS, and TS. Wrote manuscript: RV. Reviewed and approved manuscript: EP, DB, PB, JS, and TS.

### Conflict of interest statement

The authors declare that the research was conducted in the absence of any commercial or financial relationships that could be construed as a potential conflict of interest.
